# Salvinorin A Does Not Affect Seizure Threshold in Mice

**DOI:** 10.3390/molecules25051204

**Published:** 2020-03-07

**Authors:** Katarzyna Socała, Urszula Doboszewska, Piotr Wlaź

**Affiliations:** Department of Animal Physiology and Pharmacology, Maria Curie-Skłodowska University, Institute of Biological Sciences, Akademicka 19, 20-033 Lublin, Poland; urszula.doboszewska@umcs.lublin.pl

**Keywords:** salvinorin A, κ-opioid receptor, seizure threshold, anticonvulsant, proconvulsant

## Abstract

The κ-opioid receptor has recently gained attention as a new molecular target in the treatment of many psychiatric and neurological disorders including epilepsy. Salvinorin A is a potent plant-derived hallucinogen that acts as a highly selective κ-opioid receptor agonist. It has unique structure and pharmacological properties, but its influence on seizure susceptibility has not been studied so far. Therefore, the aim of the present study was to investigate the effect of salvinorin A on seizure thresholds in three acute seizure tests in mice. We also examined its effect on muscular strength and motor coordination. The obtained results showed that salvinorin A (0.1–10 mg/kg, i.p.) did not significantly affect the thresholds for the first myoclonic twitch, generalized clonic seizure, or forelimb tonus in the intravenous pentylenetetrazole seizure threshold test in mice. Likewise, it failed to affect the thresholds for tonic hindlimb extension and psychomotor seizures in the maximal electroshock- and 6 Hz-induced seizure threshold tests, respectively. Moreover, no changes in motor coordination (assessed in the chimney test) or muscular strength (assessed in the grip-strength test) were observed. This is a preliminary report only, and further studies are warranted to better characterize the effects of salvinorin A on seizure and epilepsy.

## 1. Introduction

Salvinorin A, first isolated and identified in 1984, is the main bioactive constituent responsible for the hallucinogenic properties of *Salvia divinorum* (Lamiaceae). It is a short-acting agent with relatively low toxicity [1,2]. Salvinorin A has a unique chemical structure and molecular target as compared to other naturally occurring or synthetic hallucinogens, which are mainly alkaloids acting at 5-HT_2A_ receptors. Chemically, salvinorin A is a nonnitrogenous neoclerodane diterpene [3,4]. It does not bind to serotonin receptors and is devoid of action on over fifty different receptors, transporters, and ligand-gated ion channels. The mechanism of action of this hallucinogen was unknown until 2002, when Roth and coworkers [5] demonstrated that it acts as a potent and highly selective agonist of the κ-opioid receptors. This was quite intriguing because up to then, the presence of a basic nitrogen atom was considered an absolute requirement for opioid receptor ligands. Being the first nonnitrogenous κ receptor agonist to be discovered, salvinorin A gained a lot of attention from the researchers, and much effort has been directed towards understanding the ligand–receptor interactions of salvinorin A with the target receptor and characterizing the relationship between its chemical structure and pharmacological activity [4,6].

As a highly selective κ receptor agonist, salvinorin A has become also a useful tool to study the role of the κ-opioid system [7]. A large body of evidence suggests that this receptor and its endogenous neuropeptide ligands (i.e., dynorphins) are involved in the control of motor activity, arousal, dysphoria, stress, aversion, depressive-like behavior, and drug reinstatement. Moreover, activation of the κ-opioid receptors produces analgesia, which is a well-known phenomenon [3,8]. Not surprisingly, this receptor has emerged as an important therapeutic target for the treatment of different psychiatric and neurological conditions such as mood and reward-related diseases, schizophrenia, pain, and epilepsy [8,9,10]. Pharmacological effects of salvinorin A are generally, but not always, similar to those demonstrated for other κ receptor agonists. It decreases locomotor activity and produces sedative-like effects and antinociception. Low doses of salvinorin A were shown to induce rewarding properties in rats, whereas high doses induced aversive effects [1,2,3]. Similarly to dynorphins and synthetic agonists, salvinorin A dose-dependently decreased striatal dopamine levels, and these changes were correlated with behavioral effects such as depressive-like behavior, place aversion, and decreased locomotor activity in rodents [11,12]. Interestingly, salvinorin A also produced anxiolytic- and antidepressant-like effects in rats, but only when it was administered at low doses [13]. Moreover, salvinorin A displays neuroprotective and anti-inflammatory properties and inhibits gastrointestinal motility [2].

It is noteworthy that dynorphins and κ-opioid receptors play an important role as modulators of neuronal excitability, and thereby, they are implicated in seizures and epilepsy. Generally, activation of the κ receptors with dynorphin or synthetic agonists results in anticonvulsant and antiepileptogenic effects [10,14]. Although many κ receptor-mediated effects of salvinorin A were reported, its influence on seizure susceptibility has not been studied so far. Therefore, the present study was undertaken to evaluate the effect of this compound on the seizure thresholds in three acute seizure tests in mice, namely in (1) the timed intravenous pentylenetetrazole (i.v. PTZ) seizure threshold test, (2) the maximal electroshock seizure threshold test, and (3) the 6-Hz psychomotor seizure threshold test in mice. In addition, acute adverse effects of salvinorin A on neuromuscular strength and motor coordination were investigated.

## 2. Results

### 2.1. Effect of Salvinorin A in the i.v. PTZ Seizure Threshold Test

**Figure 1:**
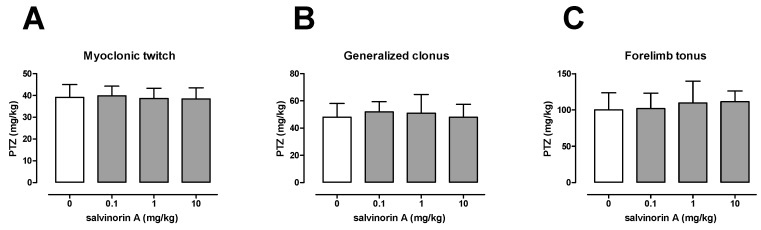
Effect of salvinorin A on the threshold for the first myoclonic twitch (**A**), generalized clonus (**B**), and forelimb tonus (**C**) in the i.v. PTZ seizure threshold test in mice. Salvinorin A was given i.p. 30 min before the test. The doses are shown on the abscissa. Control animals received 1% Tween 80. Each experimental group consisted of 9–11 animals. Data are presented as the mean (mg/kg PTZ) + SD. Statistical analysis was performed using one-way ANOVA test.

The influence of salvinorin A on the seizure threshold in the i.v. PTZ test is shown in Figure 1A–C. In the vehicle-treated group, the PTZ thresholds for the onset of the first myoclonic twitch was 39.19 ± 5.89 mg/kg, for generalized clonus was 48.06 ± 10.16 mg/kg, and for forelimb tonus was 100.2 ± 23.49 mg/kg. Salvinorin A injected at doses of 0.1, 1, and 10 mg/kg had no significant effect on any of the studied endpoints (one-way ANOVA: F(3,37) = 0.18, *p* = 0.912 for myoclonic twitch; F(3,37) = 0.38, *p* = 0.767 for generalized tonus; F(3,34) = 0.56, *p* = 0.647 for forelimb tonus).

### 2.2. Effect of Salvinorin A in the Maximal Electroshock Seizure Threshold Test

**Figure 2:**
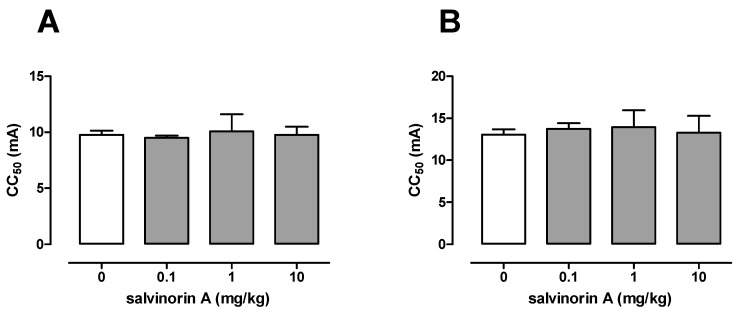
Effect of salvinorin A on the seizure threshold in the maximal electroshock seizure test (**A**) and the 6-Hz-induced seizure test (**B**) in mice. Salvinorin A was given i.p. 30 min before the test. The doses are shown on the abscissa. Control animals received 1% Tween 80. Each experimental group consisted of 20 animals. Data are presented as CC_50_ values (in mA) with upper 95% confidence limits. Each CC_50_ value represents convulsive current predicted to produce seizure in 50% of mice. Statistical analysis was performed using one-way ANOVA test.

Figure 2A presents the influence of salvinorin A on the threshold for the tonic hindlimb extension in the maximal electroshock seizure threshold test in mice. In the vehicle-treated group, the seizure threshold was 9.77 (9.41–10.15) mA. Salvinorin A administered at doses of 0.1, 1, and 10 mg/kg had no significant effect on the current intensity necessary to induce hindlimb tonus in this test (one-way ANOVA: F(3,31) = 0.33, *p* = 0.807; Figure 2).

### 2.3. Effect of Salvinorin A in the 6 Hz-Induced Seizure Threshold Test

The influence of salvinorin A on the threshold for the 6 Hz-induced psychomotor seizure is shown in Figure 2B. The control CC_50_ value for 6 Hz-induced seizure was estimated at 13.05 (12.46–13.67) mA. Salvinorin A administered at doses of 0.1, 1, and 10 mg/kg had no significant effect on the current intensity necessary to induce psychomotor seizures (one-way ANOVA: F(3,32) = 0.23, *p* = 0.877).

### 2.4. Effect of Salvinorin A on Muscular Strength and Motor Coordination

Salvinorin A (0.1–10 mg/kg) did not affect muscle strength, as determined in the grip strength test (one-way ANOVA: F(3,36) = 1.22, *p* = 0.318). Moreover, the chimney test did not reveal any impairment of motor coordination in mice (Fisher’s exact probability test: *p* = 1.000). Data from the grip strength test and chimney test are shown in Table 1.

## 3. Discussion

Salvinorin A acts as a potent and highly selective κ opioid receptors agonist. Since the dynorphin/κ opioid receptor system is involved in the neurobiology of epilepsy [10,14], there is a possibility that salvinorin A affects seizure activity. Although a wide range of pharmacological effects of salvinorin A has been demonstrated, its influence on seizure susceptibility is not known. This prompted us to investigate the effect of this unique compound on the seizure threshold in mice.

For this purpose, three different seizure threshold tests were employed in the present study. The timed i.v. PTZ test, which is considered one of the most sensitive methods for assessing seizure thresholds in rodents, was used to study the effect of salvinorin A on the thresholds for the first myoclonic twitch, generalized clonus, and tonic forelimb extension. The obtained results showed that salvinorin A did not affect any of the studied endpoints in the i.v. PTZ test. Next, the maximal electroshock- and 6 Hz-induced seizure threshold tests were used to evaluate the influence of salvinorin A on electrically induced seizures. Maximal electroshock-induced seizures are thought to mimic generalized tonic–clonic (grand mal) seizures in humans [15], whereas the 6 Hz-induced seizures model psychomotor (limbic) seizures that occur in human partial epilepsy [16]. Salvinorin A failed to affect the thresholds for both the tonic extension of the hindlimbs and psychomotor seizures in the maximal electroshock and 6 Hz seizure test, respectively. As noted above, there are no reports on the effects of salvinorin A on seizures and/or epilepsy with one exception. Listos et al. [17] mentioned that in their unpublished study, salvinorin A failed to affect pentylenetetrazole-induced seizures, even at high doses. Unfortunately, there are no more details on the study design, such as information on pretreatment time, animals used, etc.

The lack of any effect of salvinorin A on seizure threshold is a rather unexpected result because κ-opioid receptor agonists have been shown to produce anticonvulsant effects in a variety of acute seizure models in rodents including maximal electroshock- [18,19,20,21], PTZ- [22], pilocarpine- [23,24], bicuculline- [25], kainic acid- [26], and NMDA-induced [20,27] seizures. Moreover, they showed antiepileptogenic-like action in electrically- [28,29] and chemically-induced [22,29,30] kindling models. Considerable research has also focused on the role of dynorphin, an endogenous κ opioid receptor agonist, in control of neuronal excitability, seizures, epilepsy, and epileptogenesis [10,14]. Based on these reports, it appears that activation of central κ receptors with both endogenous and synthetic agonists results in anticonvulsant effects.

There could be several reasons why salvinorin A failed to affect seizure susceptibility in our study. In rodents, salvinorin A usually reaches its maximum behavioral effects 20–40 min post i.p. injection [31]. Based on a literature search, we decided to study its influence on seizure thresholds 30 min after administration. However, the dynamic positron emission tomography imaging in rats showed that salvinorin A had a prolonged effect on κ receptor binding availability long after it was eliminated from the brain [32]. Furthermore, salvinorin A produced opposite effects on intracranial self-stimulation thresholds when measured immediately or 24 h later [33]. Perhaps the 30-min pretreatment time was too short for salvinorin A to affect the κ receptor-mediated control of neuronal excitability; or, on the contrary, the effect of salvinorin A on seizure threshold was very rapid and short (i.e., it was lost within 30 min). Hence, further time-course studies are required to evaluate the effect of salvinorin A on seizure activity at different time points.

Another important consideration is that endogenous dynorphin, plant-derived salvinorin A, and synthetic κ receptor agonists may activate different signaling pathways downstream of κ receptor, leading sometimes to different physiological and/or behavioral responses. For example, salvinorin A induced robust ERK1/2 phosphorylation, which is in contrast to dynorphin and some synthetic agonists [9]. Moreover, despite high affinity towards the κ receptor, salvinorin A was about 40-fold less potent than U50,488H (a synthetic agonist) in promoting internalization of the target receptor [34]. Thus, differences in cell signaling following activation of the κ receptor with salvinorin A may, at least in part, contribute to different biological effects (including the effect on seizure susceptibility) as compared to an endogenous ligand and/or some synthetic agonists. Indeed, a number of in vitro and in vivo studies showed that salvinorin A may produce different responses than other κ receptor agonists [4]. Furthermore, it is widely accepted that salvinorin A-induced effects are mediated almost exclusively by selective binding to the κ opioid receptors. However, potential interactions of salvinorin A with other receptors have also been demonstrated. Rothman et al. [35] reported that salvinorin A partially inhibited μ-opioid receptor binding, and they suggested that it may also work as a negative allosteric modulator of μ receptors. Moreover, salvinorin A was reported to have significant affinity for the dopamine D2 receptor [36], which is in contrast to a previous report [5]. Computational studies revealed that not only κ opioid receptor, but also muscarinic acetylcholine receptor 2, cannabinoid CB1 and CB2 receptors, and the aforementioned D2 receptor, may represent potential molecular targets for salvinorin A [37]. Given the above, the differential effects of salvinorin A and standard κ-opioid receptor agonists on seizure susceptibility may result from the fact that the mechanism of action of salvinorin A may be more complex than was thought and is not limited to the activation of the κ-opioid receptors.

Salvinorin A was previously reported to produce rapid but short-lasting impairment of motor coordination, as assessed in the inverted screen [38] and the rotarod test in mice [39,40]. At lower doses (0.5–2 mg/kg), salvinorin A induced motor incoordination lasting up to 15 min [38,39], while when tested at higher doses (3–10 mg/kg), it produced motor impairment 30 min post-injection [40]. Here, we did not observe any effect of salvinorin A on motor coordination in the chimney test, even at the highest dose tested (i.e., 10 mg/kg). Likewise, salvinorin A did not affect neuromuscular strength measured in the grip-strength test.

Accumulating evidence shows that salvinorin A has a therapeutic potential for the treatment of many conditions including drug addiction, mood disorders, anxiety, schizophrenia, gastrointestinal disturbances, and pain [2,4]. But despite a number of beneficial effects, it is still questionable whether salvinorin A itself is a suitable agent for further clinical development due to its hallucinogenic properties as well as a short duration of action. Nevertheless, the unique biological effects of salvinorin A make it a widely studied compound and a very promising scaffold for the design and development of new drugs. For this reason, better pharmacological characterization of salvinorin A, including its potential effect on seizures, is highly warranted.

Importantly, seizure threshold tests allow identification of both pro- and anticonvulsant properties of the test compound. Here, salvinorin A did not produce anticonvulsant-like action, but, on the other hand, it was also devoid of proconvulsant properties. This is an important observation because there are also few reports demonstrating proconvulsant effects of κ-opioid receptor agonists [41]. What is more, *Salvia divinorum* and salvinorin A have gained popularity as recreational drugs in recent years, mainly due to the legal status in some countries and easy access through smartshops and the Internet. Their increasing usage has raised safety concerns [2,42]. 

In conclusion, the present study provides new data on pharmacological properties of salvinorin A. We showed that this compound does not affect seizure susceptibility in mice, as assessed in three acute seizure threshold tests. Our findings do not support the thesis that activation of κ opioid receptors with selective agonists result in seizure suppression. However, as mentioned above, salvinorin A has a unique pharmacological profile that can be distinct from that of other κ receptor agonists. This is a preliminary report only, and further detailed studies are required to better characterize the possible influence of salvinorin A on seizure activity.

## 4. Materials and Methods

### 4.1. Animals

Male albino Swiss mice (weighing 20–30 g) were obtained from a licensed breeder (Laboratory Animals Breeding, Ilkowice, Poland) and adapted to the laboratory conditions for at least 1 week before being used in the experiments. Animals were housed in groups of 8 per cage under controlled environmental conditions (temperature 21–24 °C, relative humidity 45%–65%) with an artificial 12/12 h light/dark cycle (light on at 6:00 a.m.) and free access to food pellets and tap water. The experiments were performed between 8:00 a.m. and 2:00 p.m. to minimize circadian influences with a minimum 30-min acclimatization period to the experimental room. Housing and experimental procedures were conducted under the guidelines provided by the European Union Directive of 22 September 2010 (2010/63/EU) and Polish legislation concerning animal experimentation. All experimental procedures were approved by the Local Ethical Committee in Lublin, Poland (license no 148/2018).

### 4.2. Treatment

Salvinorin A (Cayman Chemicals, Ann Arbor, MI, USA) was suspended in a 1% solution of Tween 80 and injected intraperitoneally (i.p.) 30 min before each test. The pretreatment time was based on literature data [31,43,44]. Control animals received vehicle only. The injection volume was 10 mL/kg.

### 4.3. Intravenous PTZ Seizure Threshold Test

Mice were restrained individually in a transparent restrainer (12 cm long, 3 cm inner diameter), and their lateral tail vein was catheterized with a 27-gauge needle attached by polyethylene tubing to a 5-mL plastic syringe containing a 1% solution of PTZ (Sigma–Aldrich, St. Louis, MO, USA). A piece of adhesive tape was used to secure the needle to the tail. The PTZ solution was infused from the syringe into the vein of a freely moving mouse at a constant rate of 0.2 mL/min using a syringe pump (Physio 22, Harvard Apparatus, Holliston, MA, USA). The time intervals from the start of PTZ infusion to the onset of the following endpoints were recorded: (1) the first myoclonic twitch, (2) generalized clonic seizure with loss of righting reflex, and (3) forelimb tonus. The PTZ infusion was stopped at the beginning of tonic seizures, which were usually lethal for mice. All surviving animals were euthanized immediately. The seizure thresholds were calculated separately for each endpoint as follows: threshold dose of PTZ (mg/kg) = infusion duration (s) × infusion rate (ml/s) × PTZ concentration (mg/mL)/body weight (kg). Data obtained in the timed i.v. PTZ seizure threshold test were presented as the amount of PTZ (in mg/kg) ± SD needed to produce the first apparent sign of each endpoint.

### 4.4. Maximal Electroshock Seizure Threshold Test

Maximal electroconvulsions were induced by applying a sinusoidal alternating current (50 Hz; duration 200 ms) via saline-soaked transcorneal electrodes using rodent shocker (type 221; Hugo Sachs Elektronik, Freiburg, Germany). During stimulation, mice were restrained manually and immediately following stimulation were placed in a transparent box for behavioral observation. Tonic extension of the hindlimbs (i.e., the rigid extension of the hindlimbs that exceeds a 90° angle with the body) was taken as the endpoint. The stimulus intensity was varied by an “up-and-down” method [45]. Each animal was stimulated only once at any given current intensity which was lowered or raised by 0.06-log intervals depending on whether the previously stimulated animal did or did not respond with the endpoint. The data obtained in groups of 20 animals were used to determine the threshold current causing hindlimb tonus in 50% of mice (CC_50_ value with confidence limits for 95% probability).

### 4.5. 6 Hz-Induced Psychomotor Seizure Threshold Test

Psychomotor seizures were induced by applying rectangular pulses (0.2 ms pulse width; 6 pulses per s) for 3 s. Stimuli were delivered via saline-soaked transcorneal electrodes using a Grass model CCU1 constant current unit coupled to a Grass S48 stimulator (Grass Technologies, Warwick, RI, USA). Mice were manually restrained during the stimulation and then released for behavioral observation. The 6 Hz-induced seizures were characterized by stunned posture, eye-blinking, head-nodding, chewing, twitching of the vibrissae, rearing, forelimb clonus, and Straub tail. Lack of the features listed above or the resumption of normal exploratory behavior within 20 s after stimulation was considered as the absence of seizures. The 6 Hz seizure threshold test was performed in groups of 20 mice stimulated with different current intensities chosen according to the “up-and-down” method described by Kimball et al. [45]. Current intensity was lowered or raised by 0.06-log intervals, depending on whether the previously stimulated animal did or did not exert tonic psychomotor seizures, respectively. The seizure threshold was expressed as the median convulsive current (CC_50_ value with confidence limits for 95% probability) predicted to produce psychomotor seizures in 50% of the animals tested.

### 4.6. Grip Strength Test

The acute effect of salvinorin A on neuromuscular strength was quantified using the grip-strength apparatus (BioSeb, Chaville, France) that consisted of a steel wire grid (8 × 8 cm) connected to the isometric force transducer. The mouse was held by the tail so that it could grasp the grid with its forepaws only. The animal was then pulled back steadily until it released the grid and the maximal force exerted by the animal just before losing grip was recorded (in newtons, N). Three measurements were taken for each mouse. The mean force was normalized to body weight and expressed in mN/g ± SD for each experimental group.

### 4.7. Chimney Test

The chimney test was used to assess the acute effect of salvinorin A on motor coordination in mice. The animals were placed individually into a transparent Plexiglas tube (inner diameter 3 cm, length 30 cm) which was horizontally located on a table. When it reached the opposite end, the tube was positioned vertically and the mouse had to climb backwards up (within 60 s) in order to escape the tube.

### 4.8. Data Analysis

Data were statistically analyzed using one-way analysis of variance (one-way ANOVA) followed by the Dunnett’s test for post-hoc comparisons. For statistical analysis of the results obtained in the MEST and the 6 Hz seizure threshold test, the CC_50_ values with 95% confidence limits were transformed into the mean value of logarithms (of current strength) with SD. Data obtained in the chimney test were analyzed by the Fisher’s exact test. The significance threshold was set at 0.05. All calculations were carried out with GraphPad Prism version 5.03 for Windows (GraphPad Software, San Diego, CA, USA).

## Figures and Tables

**Table 1:** Effect of salvinorin A on neuromuscular strength and motor coordination in mice. Salvinorin A was given i.p. 30 min before the test. Control animals received 1% Tween 80. Each experimental group consisted of 10 animals. Data are presented as mean ± SD grip strengths in millinewtons per gram of mouse body weight (mN/g) from the grip-strength test assessing skeletal muscular strength in mice and as a percentage of animals showing motor coordination impairment in the chimney test. Results from the grip-strength test were analyzed with one-way ANOVA test. Statistical analysis of data from the chimney test was performed with the Fisher’s exact probability test.

Treatment	Neuromuscular Strength (mN/g)	Impairment of Motor Coordination (%)
control	25.19 ± 3.34	0
salvinorin A 0.1 mg/kg	30.35 ± 6.49	0
salvinorin A 1 mg/kg	28.99 ± 7.27	0
salvinorin A 10 mg/kg	28.29 ± 7.15	0
